# Human decellularized dermal matrix seeded with adipose-derived stem cells enhances wound healing in a murine model: Experimental study

**DOI:** 10.1016/j.amsu.2019.07.033

**Published:** 2019-08-07

**Authors:** M. Doornaert, B. Depypere, D. Creytens, H. Declercq, J. Taminau, K. Lemeire, S. Monstrey, G. Berx, Ph. Blondeel

**Affiliations:** a Department of Plastic and Reconstructive Surgery, Gent University Hospital, Gent, Belgium; b Department of Pathology, Gent University Hospital, Gent, Belgium; c Cancer Research Institute Gent (CRIG), Gent, Belgium; d Department of Basic Medical Sciences, Ugent, Gent, Belgium; e Molecular and Cellular Oncology Laboratory, Department of Biomedical Molecular Biology, Vlaams Instituut voor Biotechnologie (VIB), Gent, Belgium; f Inflammation Research Centre (IRC), VIB, Gent, Belgium

**Keywords:** Adipose-derived stem cells, Dermal matrix, Full-thickness wounds, Mechanical, Plasma

## Abstract

**Objective:**

Full-thickness cutaneous wounds treated with split-thickness skin grafts often result in unaesthetic and hypertrophic scars. Dermal substitutes are currently used together with skin grafts in a single treatment to reconstruct the dermal layer of the skin, resulting in improved quality of scars. Adipose-derived stem cells (ASCs) have been described to enhance wound healing through structural and humoral mechanisms. In this study, we investigate the compatibility of xenogen-free isolated human ASCs seeded on human acellular dermal matrix (Glyaderm®) in a murine immunodeficient wound model.

**Methods:**

Adipose tissue was obtained from abdominal liposuction, and stromal cells were isolated mechanically and cultured xenogen-free in autologous plasma-supplemented medium. Glyaderm® discs were seeded with EGFP-transduced ASCs, and implanted on 8 mm full-thickness dorsal wounds in an immunodeficient murine model, in comparison to standard Glyaderm® discs. Re-epithelialization rate, granulation thickness and vascularity were assessed by histology on days 3, 7 and 12. Statistical analysis was conducted using the Wilcoxon signed-rank test. EGFP-staining allowed for tracking of the ASCs in vivo. Hypoxic culture of the ASCs was performed to evaluate cytokine production.

**Results:**

ASCs were characterized with flowcytometric analysis and differentiation assay. EGFP-tranduction resulted in 95% positive cells after sorting. Re-epithelialization in the ASC-seeded Glyaderm® side was significantly increased, resulting in complete wound healing in 12 days. Granulation thickness and vascularization were significantly increased during early wound healing. EGFP-ASCs could be retrieved by immunohistochemistry in the granulation tissue in early wound healing, and lining vascular structures in later stages.

**Conclusion:**

Glyaderm® is an effective carrier to deliver ASCs in full-thickness wounds. ASC-seeded Glyaderm® significantly enhances wound healing compared to standard Glyaderm®. The results of this study encourage clinical trials for treatment of full-thickness skin defects. Furthermore, xenogen-free isolation and autologous plasma-augmented culture expansion of ASCs, combined with the existing clinical experience with Glyaderm®, aid in simplifying the necessary procedures in a GMP-laboratory setting.

## Highlights

• ASCs obtained from human liposuction fat were isolated mechanically and cultured xenogen-free in autologous plasma-supplemented medium.
• Hypoxic culture of the ASCs was performed to evaluate increased cytokine production.
• Glyaderm® discs were seeded with EGFP-transduced ASCs, and implanted on full-thickness dorsal wounds in an immunodeficient murine model, in comparison to standard Glyaderm® discs.
• Reëpithelialisation, granulation and vascularization in the ASC-seeded Glyaderm® side were significantly increased, resulting in complete wound healing in 12 days.
• EGFP-ASCs could be retrieved by IHC in the granulation tissue in early wound healing, and lining vascular structures in later stages.

## Impact statement

Significantly increased wound healing was achieved by introducing ASCs seeded on human acellular dermis (Glyaderm®) into a full-thickness wound environment in a murine immunodeficient wound model, resulting in wound closure in less than 2 weeks time. This study incites clinical trials to further research this treatment in skin reconstructive surgery. Furthermore, Glyaderm® is at present commonly used in burn centers, and the completely xenogen-free isolation and autologous plasma-supplemented culture expansion of ASCs addition, described in this experiment, facilitate the use of this treatment in GMP-based laboratories.

## 1 Introduction

One of the main treatments for skin damage or absence, caused by disease or trauma, consists of split-thickness skin grafting. In case of extensive defects such as in severely burned patients, donor site morbidity limits the available thickness and surface of skin autografts, necessitating meshing and expansion of the grafts. However, it is well known that thin and widely expanded skin grafts often heal with unaesthetic and hypertrophic scars. To improve this outcome, skin substitutes such as decellularized dermis have been used to additionally reconstruct the dermal layer of the skin defect. These serve as a scaffold into which cells can migrate to form a thicker neo-dermis, and improve the quality and elasticity of tissue after wound healing [1]. Human acellular dermal matrix (ADM) allografts are considered the best skin substitutes to date [2]. They are compact and elastic, and less expensive than artificial substitutes. Glyaderm® is human allogeneic donor skin, from which all antigenic structures and cells have been removed through the use of glycerol preservation and Na–OH incubation [3]. It is provided by a non-profit skin bank and is commonly used on patients with full-thickness burns, showing engraftment after 6 days. Glyaderm® consists of a normal collagen-elastin fiber network, thus providing the optimal three-dimensional fiber structure for ingrowing fibroblasts, blood vessels or other cells seeded on it.

The reconstructive properties of adipose-derived stem cells (ASCs) have been extensively described. They are easily obtained and can be cultured xenogen-free in large amounts as previously demonstrated by our group. They have the potential to promote angiogenesis, secrete growth factors and differentiate into multiple lineages upon appropriate stimulation [4,5]. Therefore, introducing them to the local ischemic environment of the wound through an acellular matrix could effectively accelerate wound healing through promoting inflammation, granulation and neovascularization.

Besides this indirect paracrine effect, direct enhancement of wound healing through differentiation into endothelial and epithelial lineages has been described [[6], [7], [8]].

In this study, we investigated an acellular human dermal matrix seeded with human ASCs in a severely combined immunodeficient (SCID) murine model as a proof of principle for treatment of full-thickness skin defects.

## 2 Materials and methods

Adipose tissue was obtained from an abdominal liposuction procedure in a healthy female patient (age 36 years) after obtaining informed consent using an UZ Gent ethical review board-approved protocol. After infiltration with Klein's solution, a 3-mm blunt cannula (Mentor, Santa Barbara, Cal, USA) was used in combination with device aspiration at −1,5 atm. 50 cc of peripheral venous blood was also obtained during the procedure by venous puncture.

For isolation of ASC's from the lipo-aspirate, a previously described protocol was used. In brief, the lipo-aspirate was centrifuged in 10 cc luer-lok™ syringes (Becton Dickinson, Franklin Lake, NJ, USA) at 3000 rpm for 1 min. After centrifugation, the oily and fluid phases were discarded, and the fat was transferred to produce 20 cc syringes filled with exactly 10 cc centrifuged fat. Next, a 20 cc syringe filled with 10 cc centrifuged lipo-aspirate was connected luer-to-luer with another 20 cc syringe filled with 10 cc PBS (phosphate-buffered saline), and the contents were forcefully pushed back-and-forth 30 times. The resulting PBS-diluted adipose emulsion was then centrifuged again for 10 min at 3000 rpm.

The blood samples were processed to extract plasma. After centrifugation for 10 min at 4000 rpm, the supernatant fluid layer without the buffy coat was obtained. The plasma was then passed through a 0,22 μm filter. Culture medium was created with 90% DMEM-hepes (Sigma-Aldrich, St Louis, MO, USA), 10% human plasma, 1% Penicillin/Streptomycin (Sigma-Aldrich, St Louis, MO, USA) and 100 IU of heparin (Leo Pharma, Lier, Belgium). After 48 h, the medium was replaced to sort the cells for adherence. After 3 more days, the medium was replaced again.

After 8 days, cells were released using TrypLE Select (Thermo- Fisher, Waltham MA, USA). The P1 viable cells were counted using propidium iodide (Sigma-Aldrich, St Louis, MO, USA) and suspended in PBS for flowcytometric analysis.

### 2.1 Flow cytometry

First-passage ASCs were labeled with CD13-APC-Vio700, CD31-APC, CD34-PE, CD45-VioGreen, CD73-PE-Vio770, CD105-VioBlue and CD146-VioBright515, purchased from Miltenyi Biotec (Bergisch Gladbach, Germany). Samples were acquired on a four-laser flow cytometry system (BD LSR II, Becton-Dickinson, Franklin Lake, NJ, USA). Fc receptor reagent was added to avoid unspecific labelling of cells via Fc receptors.

### 2.2 Differentiation assay

First-passage ASCs were replated on thermanox cover slips (Nunc, Roskilde, Denmark) for adipogenic differentiation, and another part for osteogenic differentiation. Adipogenic differentiation was performed using basic culture medium with addition of dexamethasone, insuline, indomethacine and 3-isobutyl-1-methylxanthine (Sigma-Aldrich, St Louis, MO, USA). Differentiation was confirmed by Oil Red O stain. Osteogenic differentiation was performed using basic culture medium with addition of dexamethasone, ascorbic acid and β-glycerophosphate (Sigma-Aldrich, St Louis, MO, USA). Differentiation was confirmed by Von Kossa's Stain. Digital imaging of all wells was performed using an Olympus inverted microscope, and Cell^M software (Olympus Europe, Hamburg, Germany).

### 2.3 EGFP-transduction of ASCs

293T cells were cultured in DMEM (41965039, ThermoFisher) with 10% Fetal Calf Serum (FCS) (Sigma-Aldrich, St Louis, MO, USA) and 2 mM l-Glutamine (BE17-605F, Lonza) and transfected with lentiviral envelope plasmid pMD2.G, packaging plasmid psPAX2 and lentiviral EGFP expression plasmid pLenti6-EGFP. The medium was removed and replaced with fresh medium 8 h post transfection. The virus was harvested 48 h post-transfection and filtered through a 0.45 μm PES filter (Merck- Millipore, Burlington, Massachusetts, USA). P1 ASC's were cultured in DMEM with 10% FCS and 2 mM l-Glutamine until a density of approximately 60% was reached. The medium was removed and replaced by pLenti6-EGFP virus containing medium for 24 h. After 10 days the EGFP positive cells were sorted with the BD FACSAria III cell sorter.

### 2.4 Hypoxic culture of ASCs

Second passage ASCs were cultured for 7 days in normal conditions, and compared to second passage ASCs cultured in hypoxic conditions with 5% O2 and 5% CO2 in the incubator. After 4 days and 7 days, the culture medium was collected and compared to base line culture medium. The samples were shipped on dry ice to Eve Technologies, Calgary, Canada for cytokine array on the following cytokines: Insulin binding growth factor binding protein-1 (IGFBP1), Epidermal growth Factor (EGF), fibroblast growth factor-1(FGF-1), fibroblast growth factor-2 (FGF-2), Granulocyte colony stimulating factor (G-CSF), Eotaxin-1.

Tumour growth factor-α (TGF-α), Platelet Derived Growth Factor-α (PDGF-α), Platelet Derived Growth Factor-β (PDGF-β), Interleukine-1B (IL-1B), Interleukine-2 (IL-2), Interleukine-3 (IL-3), Interleukine-9 (IL-9), Interleukine-10 (IL-10), Interleukine-13 (IL-13).

Vascular Endothelial Growth Factor-A (VEGF-A), Tumour Necrosis Factor-α (TNF-α), Fibroblast Growth Factor-1 (FGF-1), Fibroblast Growth Factor-2 (FGF-2), Granulocyte-Macrophage Colony Stimulating Factor (GM-CSF), Growth-Related Oncogene-α (GRO-α), FMS-like Tyrosine Kinase 3L (Flt-3L).

### 2.5 Compatibility assay: preparation of Glyaderm® in vitro

Glyaderm® is a 0,3 mm dermal substitute derived from glycerol-preserved human allogeneic skin. Samples were rinsed three times in PBS, after which an 8 mm punch biopsy device (Kai Medical, Seki, Japan) was used to create discs. The Glyaderm® discs were placed in a 48-well plate (ThermoFischer Scientific, MA, US) after which 0,2 ml of medium containing 10^5^ EGFP-ASCs was added. After 4 h, extra medium was added to 1 ml. GFP-ASCs were cultured on the Glyaderm® for 12 days as a pre-study. After 3, 7 and 12 days, the Glyaderm® discs containing the GFP-ASCs were fixed in 4% formalin for at least 24 h at 5 °C and subsequently embedded in paraffin and sectioned at 5 μm.

### 2.6 *In vivo* analysis of effect of ASC-seeded Glyaderm® versus standard Glyaderm® on full-thickness wound murine model

Preparation of the Glyaderm® was identical as described above in the in vitro compatibility assay: 15 Glyaderm® discs seeded with 10^5^ EGFP-ASCs and 15 Glyaderm® discs were kept in 48-well plates for 5 days before the animal experiment.

15 female eight-week-old T-cell deficient nude mice (BALB/c-nude; Envigo, Huntingdon, UK) were used in this study. The study was approved by the Ghent University Hospital Ethical Committee and all animal experiments were conducted according to this institutions guidelines, and compliant to the ARRIVE criteria [9]. For the sample size, we based our calculation on similar research [10], but limited the events of experimental data collection to reduce the number of animal test subjects. All of the surgical instruments were sterilized, and surgical procedures were performed under laminar flow. The surgical sites on the mouse skin were sterilized with Chlorhexidin digluconate 0,5% in aqua. Animal anaesthesia was achieved with isoflurane vaporizer 3,5% for induction, and 1% for maintenance, with non-rebreather mask. After anaesthesia, a rounded, full-thickness, 8-mm cutaneous wound was made by punch biopsy instrument on each mid-dorsum by lifting the dorsal skin in a fold and pushing the punch biopt through and through against a sterile compress, so the full-thickness aspect of the wound was assured. The left full-thickness wound was covered with an 8 mm Glyaderm® disc. The right full-thickness wound was covered with an 8 mm Glyaderm® disc seeded with EGFP-positive ASCs, with the cell-seeded side facing the wound. The discs were fixed with 3 sutures vicryl 5/0 (Johnson&Johnson, New Jersey, US) and covered with Tegaderm® film (3 M Health, MN, US), to prevent contraction and dehydration. The peri- and post-intervention period was uneventful. The mice were housed in groups of five in specific pathogen-free (SPF) rooms and micro-isolator cages. They were provided with sterile nesting material and SC Ketoprofen (Novartis, Basel, Switzerland) for post-operative pain relief upon signs of distress [11]. To evaluate the wound healing and ingrowth of the Glyaderm®, the mice were sacrificed on postoperative day 3 (n = 5), day 7 (n = 5) and day 12 (n = 5) by isoflurane anaesthesia induction followed by cervical dislocation. The grafts with surrounding skin and upper dorsal muscle layer were recovered by sharp dissection. Digital microscopy imaging (Dino-lite, Naarden, The Netherlands) of the wounds was performed for evaluation of re-epithelialization. Two-ruler wound area measurement and planimetry (ImageJ [12]) were performed, as described in detail by Foltynksi et al. [13]. In brief, a straight line segment was drawn at the scale bar, “Set Scale” from the “Analyse” menu was chosen and the number of pixels from the field “Distance in pixels” was noted, corresponding now with the length of the scale bar. Next, using the “Measurement” tool, a polygon was drawn along the remaining wound surface. This value was normalized to the 50,3 mm^3^ original wound surface of the 8 mm Glyaderm® discs.

### 2.7 Histology

5-μm paraffin sections of the skin grafts were made through the center and perpendicular to the surface of the wound. The sections were stained with H&E and photographed by light microscopy (Olympus BX50, Hamburg, Germany). Additional Immunohistochemistry (IHC) staining for Smooth Muscle Actin (SMA) (clone 1A4, Dako/Agilent, Santa Clara, US), CD31 (clone JC70A, Dako/Agilent, Santa Clara, US) and Ets-Related Gene (ERG) (clone EPR3864, Roche, Vilvoorde, Belgium) was performed.

Additionally, sections were examined with GFP staining: tissue sections were deparaffinised and rehydrated. Antigen retrieval was performed by heating the sections in 10 mM sodium citrate buffer (pH 6.0) (Vector laboratories, Burlingame, US) in an electric pressure cooker. Blocking of endogenous peroxidase occurred in 3% H2O2 in methanol. Sections were then treated with 5% goat serum in PBS+1% BSA, followed by an overnight incubation with primary GFP-antibody (Clone D5.1, Cell Signaling Technology, US) at 4 °C. Detection was done with a biotin-conjugated secondary antibody followed by Avidin-Biotin complex (ABC) and developed with diaminobenzidine (DAB) (Vector Laboratories, Burlingame, US). Stained sections were analysed by light microscopy (Olympus BX50, Hamburg, Germany).

For each section, the granulation thickness and blood vessel density were evaluated. The granulation thickness at 3 points (centre of the remaining Glyaderm, and 2 points exactly between the centre and the edge of the Glyaderm®) was measured on H&E and SMA stained sections (magnification 400x). For assessment of blood vessel density, randomized areas of ERG-stained sections (magnification 400x, 1 high-power field) were photographed, and vessels containing ERG-stained endothelial cells were counted. The number of blood vessels was quantified across 4 non-consecutive high-power field areas for each wound.

### 2.8 Statistical analysis

All quantitative data are expressed as mean ± standard error of the mean. Statistical analysis was conducted with the Wilcoxon signed-rank test. All tests were performed by commercially-available statistical software SPSS Statistics version 25 (IBM, New York, US). A result was deemed statistically significant if the probability was lower than 5% or a P-value<0,05.

## 3 Results

### 3.1 Characterization of the human ASCs and EGFP-transduction

**Fig. 1:**
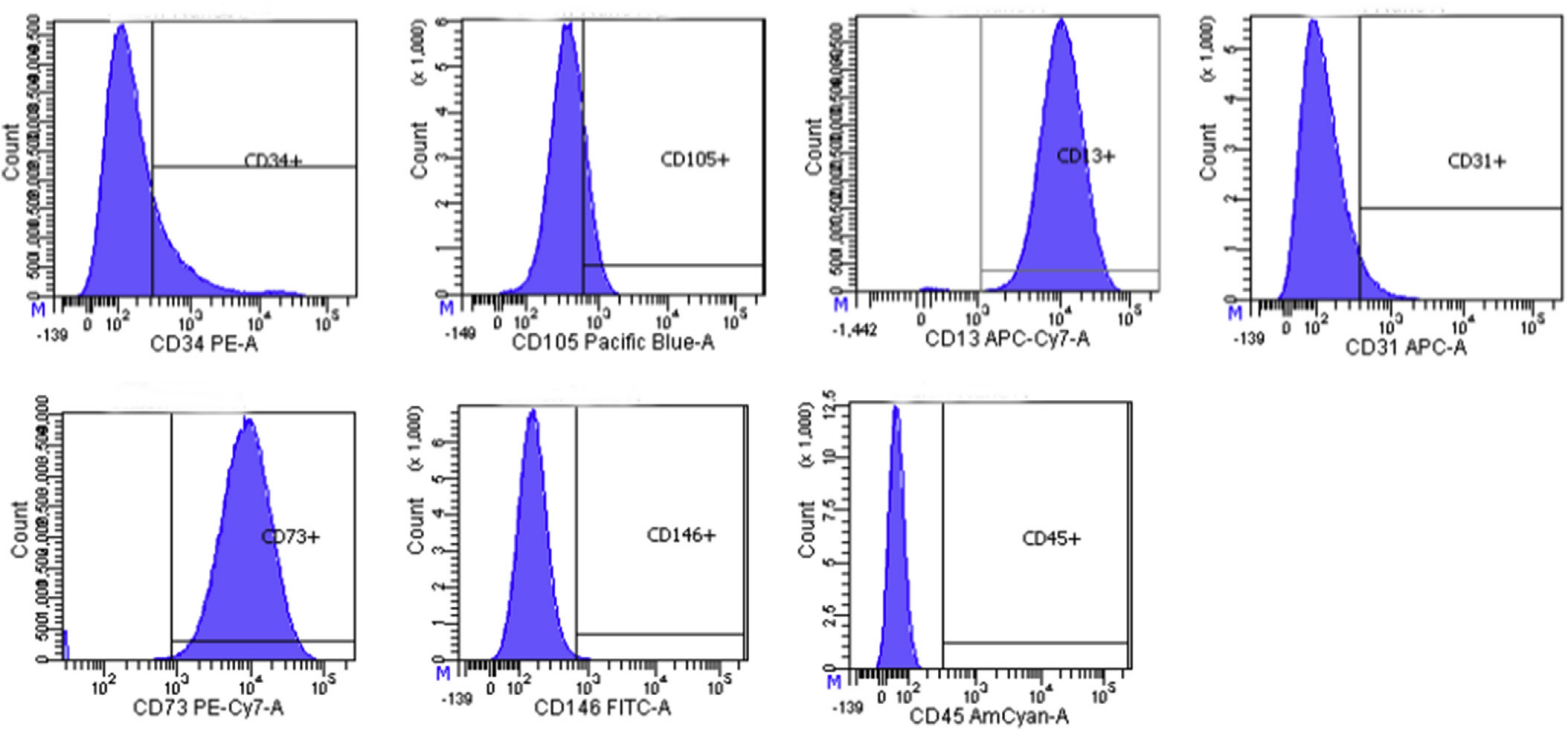
Representative histogram of ASC marker analysis; Cell surface markers were analysed by flow cytometry for the expression of CD34, CD73, CD13, CD105, CD45, CD31 and CD146.

**Fig. 2:**
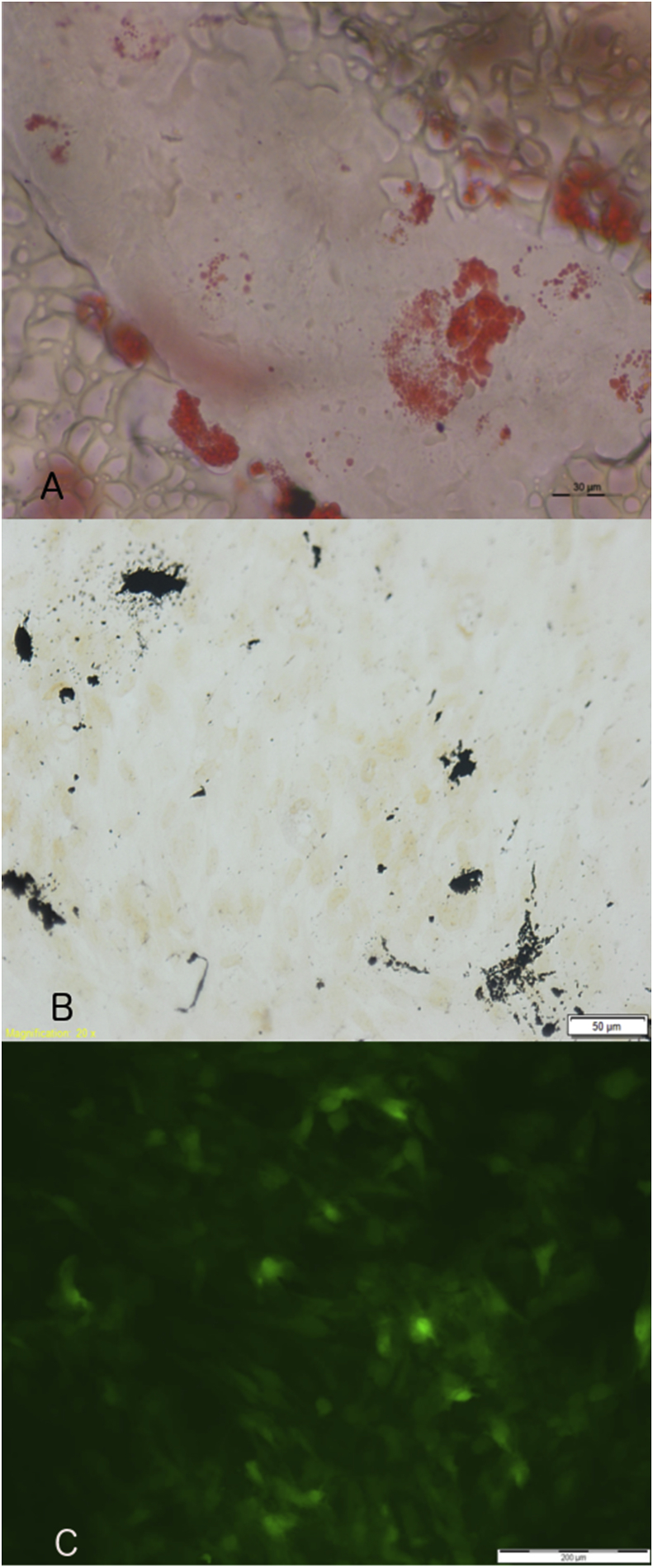
Differentiation assay. A) ASCs were assessed for adipogenic differentiation and stained with Oil red O. B) ASCs were assessed for osteogenic differentiation and stained with Von Kossa's stain. C) ASCs were transfected with pLenti6-EGFP, unstained image under fluorescence microscope. (For interpretation of the references to colour in this figure legend, the reader is referred to the Web version of this article.)

To determine the presence of ASCs in the mechanically isolated stromal cells from the human fat, flow cytometry using markers for ASCs as suggested by recent literature was performed [14,15]. First passage ASCs contained 46,8% CD13, 46,9% CD73, 9,8% CD34, 6,7% CD105 and 2,7% CD31, 0,1% CD45 and 0,5% CD146 (Fig. 1). By the fourth passage, CD34 had disappeared. Differentiation assays confirmed multilineage potential into adipogenic (Fig. 2A) and osteogenic lineage (Fig. 2B). To enable tracking of the ASCs during the in vivo experiment, EGFP-transduction of the ASCs was performed and resulted in 45,3% EGFP-positive ASCs before sorting, and 95% after sorting (Fig. 2C).

### 3.2 Cytokine array

Results from the cytokine array are listed in Table 1. Some growth factors are present in the basic culture medium, containing 10% human plasma. All growth factors are strongly upregulated in hypoxic culture conditions compared to normal culture conditions.

**Table 1:** Comparison ASC culture cytokine array in normal and hypoxic culture environment.

Cytokine	ASC D2	medium	D4	hypox D4	D7	hypox D7
IGFBP1	0,03	0,1	0,31	0,93	2,78	8,33
EGF	0	3,5	15,8	80,26	402,87	1848,43
FGF-2	0	26,32	77,54	405,39	1949,56	13089,05
Eotaxin-1	3,2	16	80	400	2000	6783,95
TGF-a	0,63	3,33	15,32	82,18	404,73	1463,37
G-CSF	0,29	3,98	15,44	78,44	411,42	1955,39
Flt-3L	0,51	3,41	15,52	82,18	390,41	2126,12
GM-CSF	0,64	3,2	16	80	400	2000
GRO-alpha	0	0	21,76	78,03	403,82	1985,26
IL-10	0,64	3,28	15,34	83,33	392,34	2018,46
PDGF-AA	0,62	3,28	16,61	76,66	425,41	2159,53
PDGF-BB	0,55	3,48	15,49	80,8	399,7	1999,57
IL-13	0,64	3,24	15,35	84,68	388,92	2084,85
IL-9	0,65	3,05	16,59	82,64	365,66	3247,21
IL-1B	0,65	3,1	16,12	82,47	385,34	2103,1
IL-2	0,65	3,13	15,03	92,62	372,14	2185,38
IL-3	0,61	3,41	15,44	79	427,69	1797,25
TNF-@	0,062	3,37	15,11	84,22	382,71	3035,84
VEGF-A	0	4,57	15,51	80,57	399,12	2006,8
EGF	0,9	2,82	8,11	24,46	76,32	215,04
FGF-1	4,57	13,72	41,15	123,46	370,37	1111,11
FGF-2	17,65	39,35	123,61	375,26	1063,62	4471,22

Cytokine array, Values are Pg/ml. D2: P1 ASCs 24 h after adherence (Medium: 90% DMEM-hepes, 10% human plasma, 1% P/S and 100 IU of heparin); D4: P1 ASCs day 4 in culture; D4 hypox: ASCs day 4 in culture in hypoxic culture conditions (5% O2, 5% CO2); D7: P1 ASCs day 7 in culture; D7 hypox: ASCs day 7 in culture in hypoxic culture conditions (5% O2, 5% CO2).

Abbreviations: adipose-derived stem cells (ASC), growth factors in basic medium (90% DMEM-hepes, 10% human plasma, 1% P/S and 100 IU of heparin) day 2 of culture (ASC D2), day 4 of culture (D4), day 4 of culture in hypoxic environment (Hypox D4), day 7 of culture (D7), day 7 of culture in hypoxic environment (Hypox D7), Insulin binding growth factor binding protein-1 (IGFBP1), Epidermal growth Factor (EGF), fibroblast growth factor-1(FGF-1), fibroblast growth factor-2 (FGF-2), Granulocyte colony stimulating factor (G-CSF), Eotaxin-1, Tumour growth factor-α (TGF-α), Platelet Derived Growth Factor-α (PDGF-α), Platelet Derived Growth Factor-β (PDGF-β), Interleukine-1B (IL-1B), Interleukine-2 (IL-2), Interleukine-3 (IL-3), Interleukine-9 (IL-9), Interleukine-10 (IL-10), Interleukine-13 (IL-13), Vascular Endothelial Growth Factor-A (VEGF-A), Tumour Necrosis Factor-α (TNF-α), Fibroblast Growth Factor-1 (FGF-1), Fibroblast Growth Factor-2 (FGF-2), Granulocyte-Macrophage Colony Stimulating Factor (GM-CSF), Growth-Related Oncogene-α (GRO-α), FMS-like Tyrosine Kinase 3L (Flt-3L).

### 3.3 Preparation of Glyaderm® in vitro

**Fig. 3:**
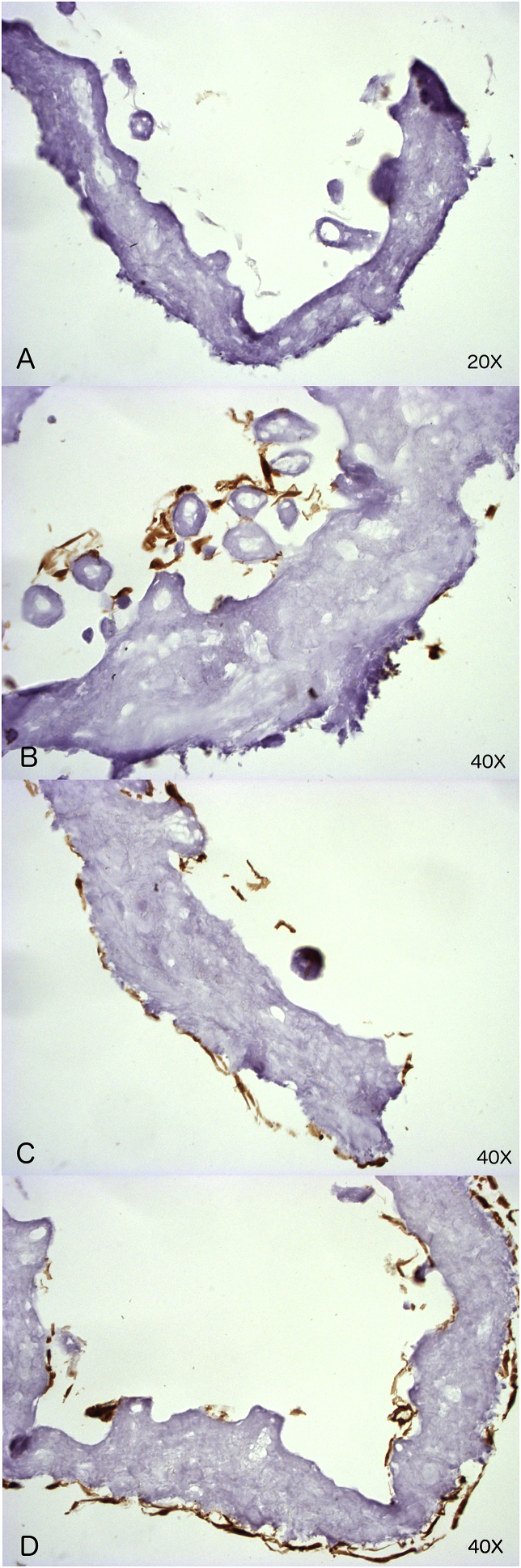
Histological results for EGFP-ASCs. A–D: Compatibility assay for 10^4^ EGFP-ASCs seeded on 8 mm Glyaderm® patch in vitro. IHC staining for EGFP-expression in ochre. A) Control Glyaderm® (10x). B) ASCs seeded Glyaderm® day 3 (40x). C) ASCs seeded Glyaderm® day 7 (20x). D) ASCs seeded Glyaderm® day 12 (20x). E–F: In vivo experiment. IHC-staining of EGFP-ASCs in ochre. E) EGFP-ASCs are stained at the undersurface of the Glyaderm® on day 3 (10x). F) EGFP-ASCs are stained at the undersurface of the Glyaderm® on day 7 (20x). G) Detail image of EGFP-ASCs on day 7 (40x). H) EGFP-ASCs are retrieved in the granulation tissue on day 12 (20x).

EGFP-positive cells were visualised by IHC, attached to the upper surface of the Glyaderm® discs. At day 3 the cells were dispersed, but were observed to progressively cover the surface of the Glyaderm® by day 7, and confluency further increased by day 12 (Fig. 3).

### 3.4 *In vivo* analysis of effect of ASC-seeded glyaderm® versus standard glyaderm® on a full-thickness wound in a murine model

All experimental procedures were well tolerated by the murine model. To compare the effect of the human ASCs-seeded Glyaderm® versus the standard Glyaderm® on a full-thickness dorsal wound, 5 mice were sacrificed on post-operative day 3, 5 and 12.

**Fig. 4:**
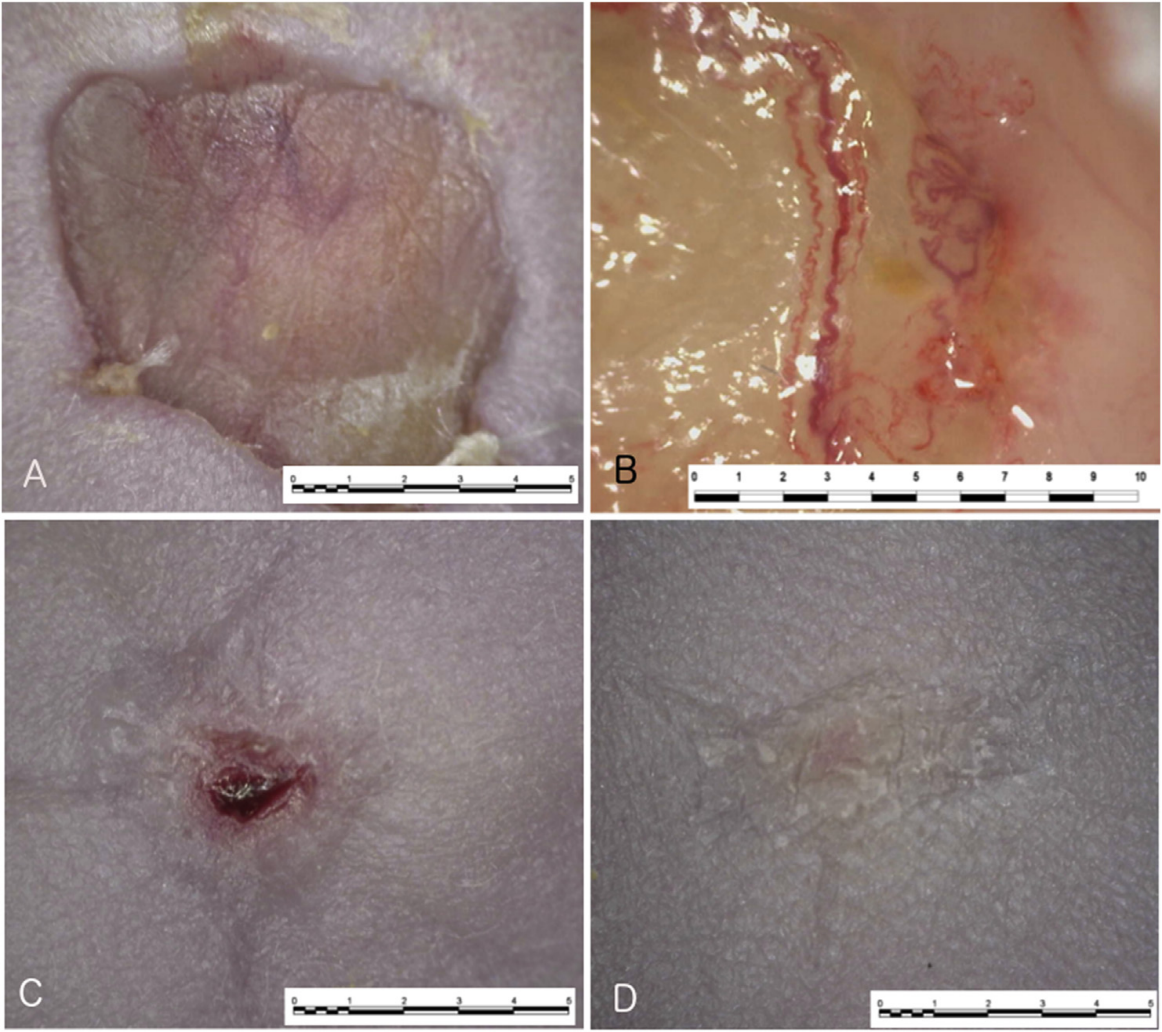
Digital microscopy of in vivo murine full-thickness wound healing model. A) Glyaderm® day 3. B) ASC-seeded Glyaderm® day 3. C) normal Glyaderm® day 7. D) ASC-seeded Glyaderm® day 7. E) Glyaderm® day 12. F) ASC-seeded Glyaderm® day 12.

On post-operative day 3, wound areas measured by digital planimetry showed increased re-epithelialization on the right side of compared to the left side (30,6 ± 15,6 vs. 20 ± 7%; P = 0,068). On day 7, the re-epithelialized area on the right side again was larger (43,8 ± 5, vs. 34,2 ± 2,3%; P = 0,0039). On day 12, re-epithelialization on the right side was again significantly increased (87,7 ± 1,5 vs. 75,5 ± 5,5%; P = 0,0046). Digital photography shows the wounds on the right side achieving near complete re-epithelialization, in contrast to the left side (Fig. 4).

**Fig. 5:**
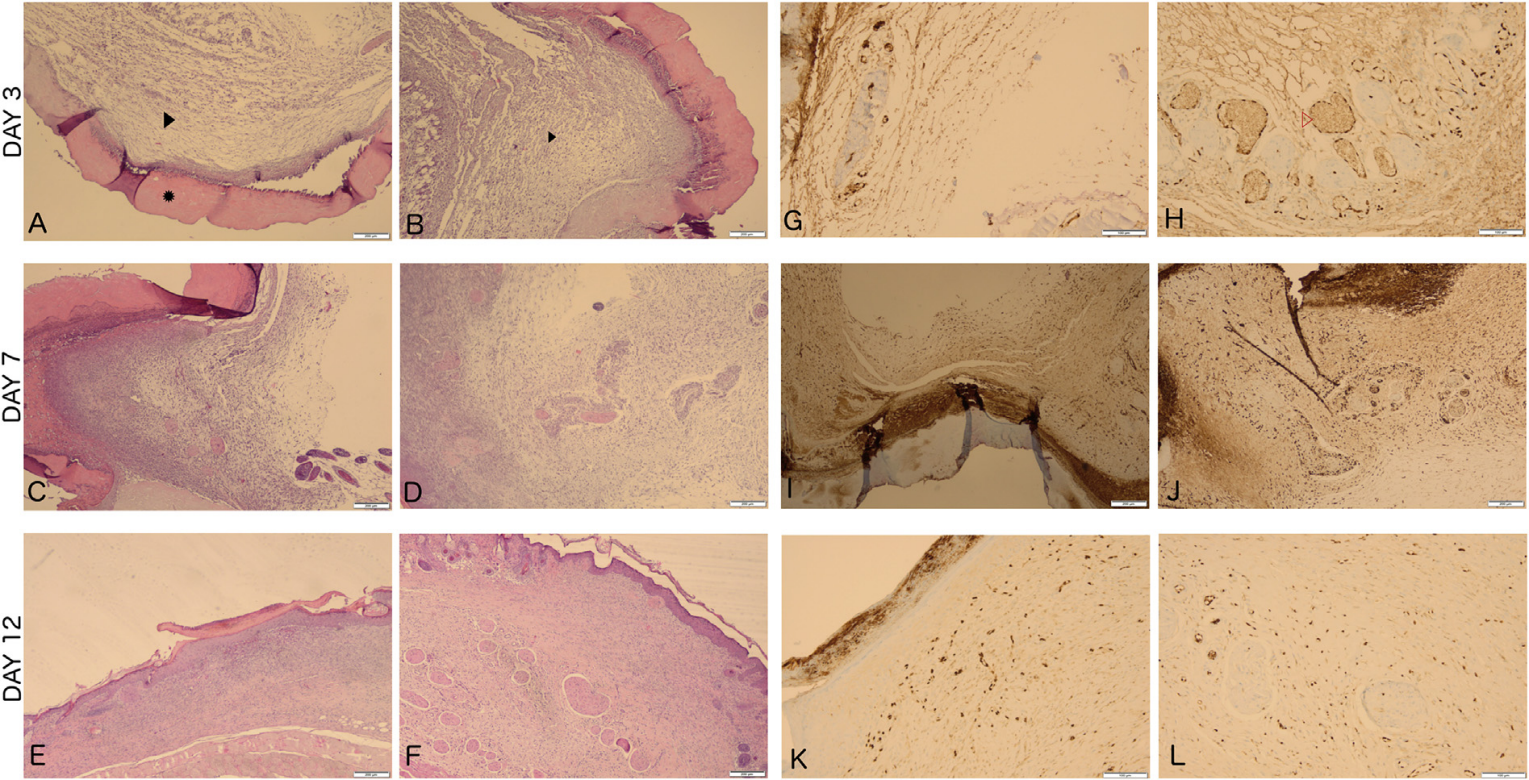
Histological results for wound healing. * = Glyaderm®, ▸ = granulation tissue, ▷ = ERG-staining of endothelial cells Right panel. (A-F) H&E staining; A) Left side, day 3. B) Right side, day 3. C) Left side, day 7. D) Right side, day 7. E) Left side, day 12. F) Right side, day 12. Left panel (G–L) ERG staining. G) Left side, day 3. H) Right side, day 3. I) Left side, day 7. J) Right side, day 7. K) Left side, day 12. L) Right side, day 12.

Granulation tissue under the Glyaderm® patch was significantly increased in all right side specimen on day 3 compared to the left side (0,87 ± 0,34 vs. 0,52 ± 0,17 mm; P = 0,043). On day 7, there was significantly more granulation thickness on the right side compared to left (134 ± 49,3 vs. 76 ± 26 mm; P = 0,043). On day 12 however, a trend towards more granulation tissue was seen on the left side compared to the right (87,5 ± 2,1 vs. 92,5 ± 30,5 mm, P = 0,068) (Fig. 5).

There was a significantly larger number of newly formed capillaries under the Glyaderm® patch on the right side compared to the left on day 3 (5,5 ± 0,15 vs. 2,34 ± 0,89 per HPF; P = 0,043), and on day 7 (21,1 ± 25,3 vs. 7,3 ± 0,29 per HPF; P = 0,043). Macroscopically, neovascularization with even vascular pedicles consisting of 2 veins and one artery growing into the Glyaderm® patch were observed (Fig. 4). On day 12, a trend towards more vascularization was seen on the left side (11,6 ± 3,1 vs. 16,7 ± 0,14 per HPF; P = 0,066) (Fig. 5).

The EGFP-ASCs could be retrieved by GFP staining on the undersurface of the Glyaderm® on the right side on days 3 and 7, and in some specimen lining the lumen of blood vessel walls in the granulation tissue on day 12 (Fig. 3).

## 4 Discussion

In this study, Glyaderm® human acellular dermal matrix demonstrated to be an efficient carrier for human ASCs. Glyaderm® is currently clinically applied in full-thickness wounds such as deep burns, in co-application with thin split-thickness skin grafts. Loose-skin animals such as mice heal 90% of their wounds by contraction, unlike humans [16]. However, dermal substitutes, especially acellular dermal matrix have shown to decrease contraction, in particular when seeded with fibroblasts or SVF cells [[17], [18], [19], [20]]. The dermal matrix provides a structural support withstanding contraction. In another explanation, the myofibroblasts that are responsible for contraction disappear when epithelialization is complete, hence faster epidermis regeneration suppresses wound contraction [21]. More recently, exosomes from ASC have been described preventing differentiation from fibroblasts into myofibroblasts and preventing granulation tissue [22]. In our in vivo experiment, Glyaderm® was used as a vector for efficient ASC delivery to a full-thickness murine wound bed. Although Glyaderm® is thin (0,3 mm) and pliable, which aids in epithelial overgrowth in this murine model, significantly accelerated healing was observed with ASC-seeded Glyaderm® in comparison to normal Glyaderm®-treated wounds. The histologic results demonstrate an augmented inflammatory phase with significantly more granulation tissue and vascularization on the ASC side during day 3 till day 7. However, on day 12, the situation seems reversed, although not significant. This could indicate that the inflammatory and proliferative phases on the ASC augmented side are already reaching the next step of wound healing, the remodeling phase. This corroborates the clinical images of the ASC-augmented wounds on the right sides who appear re-epithelialized and lack the induration still present on the left sides. Since time to healing is a predominant factor in the degree of scarring, this finding is of clinical relevance.

Our in vitro cultures confirm that ASCs in hypoxic environment are capable of increased production of numerous growth factors, contributing in the inflammatory and proliferative phases of wound healing. In pathophysiology, platelets release cytokines attracting neutrophils and macrophages that migrate over the fibrin gel cloth. Macrophages produce VEGF and TGF-β, IL-1, IL-6 and TNF-α [23]. Numerous other studies have demonstrated the paracrine wound healing effects of ASCs [4,[24], [25], [26], [27]]. Simultaneously, fibroblastic or mesenchymal cells proliferate to produce extracellular matrix (ECM) and endothelial cells (ECs) creating new blood vessels. Besides the paracrine effect, the GFP-marked ASCs in this experiment could be retrieved in vivo on the underside of the Glyaderm® in the granulation tissue, and in later time points, lining blood vessels in the granulation tissue. These findings corroborate those of Sivan et al. who have demonstrated the differentiation of ASCs into dermal-like fibroblasts and the deposition of dermal-specific ECM by these cells [28] and of others have described incorporation of ASCs into blood vessel walls [10]. Microscopically, ingrowth of neovascular structures into Glyaderm® was observed.

Other researchers have performed similar experiments as ours. Foubert et al. compared Integra® seeded with porcine ASCs to normal Integra® and found increased neovascularization and collagen deposition in the ASC group [29]. Hendrickx et al. have successfully used blood outgrowth endothelial cells (BOECs) on multilayered fibroblast sheets in full-thickness wounds to promote revascularization and inhibit wound contraction [30]. However, in the adult patient the relative scarcity of BOECs, even with culture expansion, would require large volume flebotomies. Nambu et al. found increased granulation and revascularization when using murine ASCs combined with atelocollagen matrix in diabetic wounds [31]. Huang et al. also used a mouse model with murine acellular dermal matrix and ASCs to prove enhanced wound healing, granulation and revascularization [10]. Nie et al. found that rat ASCs delivered by an Alloderm® matrix accelerated diabetic wound healing [32]. Our results corroborate their findings.

In our study, a T-cell deficient nude mouse strain was used. The murine epidermis houses populations of high and low self-renewing epidermal stem cells for short- and long-term wound repair, that might not occur in human tissues [33]. On the other hand, impaired wound healing in T-cell deficient mice has been extensively described [[34], [35], [36], [37], [38]], with decreased granulation and decreased wound bed vascularization. Although animal models have limited correlation to human wounds, our model allowed to test the efficacy of human acellular dermal matrix as a carrier for human ASCs, that were isolated and cultured xenogen-free, in vivo. Both are currently already used separately in the clinical practice, and the beneficial combination described in this study may thus encourage future clinical trials with autologous ASC-seeded dermal matrix. A cGMP-facility would allow for rapid expansion and clinical use of the ASCs on Glyaderm®. The harvest of human ASCs through small-volume liposuction would thus not significantly compromise systemic physiology in patients already under anaesthesia for e.g. early burn debridement.

In conclusion, Glyaderm® is an effective carrier for human ASCs. In this immunodeficient murine model, the combination of ASC-seeded acellular dermal matrix allowed for enhanced wound healing, both through paracrine and histological structural support. Based on these results, this study encourages future clinical trials to elaborate this treatment for full-thickness skin defects.

## Provenance and peer review

Not commissioned, internally reviewed.

## Ethical Approval

Ethical Approval was provided by the Ghent University Hospital Ethische Commissie Dierproeven (ECD), reference number LA1400072.

Under the number ECD 17/39; All animal experiments were conducted according to this institutions guidelines.

## Sources of funding

Nothing to declare.

## Author contribution

Maarten AJ Doornaert: Experimental setup, laboratory experiments, draft writing.

Bernard Depypere: Laboratory experiments, animal experiment.

David Creytens: histological analysis, data collection.

Joachim Taminau: cell culture, data collection.

Kelly Lemeire: data collection, histology.

Heidi Declercq: Laboratory experiments, cell culture, review.

Geert Berx: theoretical support, study design, review.

Phillip Blondeel: writing, theoretical discussion, review.

## Conflicts of interest

Nothing to declare.

## Guarantor

Maarten Doornaert, MD, PhD.
